# PNI as a Potential Add-On Biomarker to Improve the IMDC Intermediate Prognostic Score

**DOI:** 10.3390/jcm12196420

**Published:** 2023-10-09

**Authors:** İbrahim Vedat Bayoğlu, Javid Hüseynov, Alper Topal, Nadiye Sever, Nargiz Majidova, Abdussamet Çelebi, Alper Yaşar, Rukiye Arıkan, Selver Işık, Muhammet Bekir Hacıoğlu, Özlem Ercelep, Murat Sarı, Bülent Erdoğan, İlhan Hacıbekiroğlu, Sernaz Topaloğlu, Osman Köstek, İrfan Çiçin

**Affiliations:** 1 Department of Medical Oncology, School of Medicine, Marmara University, 34899 Istanbul, Turkey; cavidmurad1793@gmail.com (J.H.);; 2 Department of Medical Oncology, School of Medicine, Trakya University, 22000 Edirne, Turkey; alpertsroom@gmail.com (A.T.);; 3 Department of Medical Oncology, School of Medicine, Sakarya University, 54290 Sakarya, Turkey

**Keywords:** PNI, IMDC model, prognosis, metastatic RCC, adjusted PNI-IMDC model

## Abstract

Introduction: This study aimed to assess the role of the adjusted PNI-IMDC risk scoring system in stratifying the intermediate group of metastatic RCC patients who received TKIS in the first-line setting. Methods: A total of 185 patients were included. The adjusted PNI and IMDC model was used to divide the intermediate group into two groups: intermediate PNI-high and intermediate PNI-low groups. The statistical data were analyzed using Kaplan–Meier and Cox regression analysis. Results: The results showed that the adjusted PNI-IMDC risk score, classic IMDC, and PNI had similar prognostic values. Adjusted PNI-IMDC risk score might be used for a more homogeneous differentiation of the classic intermediate group. On the other hand, multivariate analysis revealed that the presence of nephrectomy, adjusted favorable/intermediate (PNI-high) group, ECOG performance score, and presence of bone metastasis were independent predictors of OS. Conclusions: Pre-treatment PNI, as a valuable and potential add-on biomarker to the adjusted PNI-IMDC classification model, can be helpful for establishing an improved prognostic model for intermediate group mRCC patients treated with first-line TKISs. Further validation studies are needed to clarify these findings.

## 1. Introduction

Kidney cancer accounts for about 2% of malignancies worldwide and has a heterogenous biology. Its incidence has been rising in younger individuals [1]. Renal cell carcinoma (RCC) comprises approximately 4.1% of all new cancers, with a median age at diagnosis of 64 years. Approximately 85% of kidney tumors are RCC, and approximately 70% of these have a clear cell RCC. Other less common cell types include papillary, chromophobe, translocation, and Bellini duct (collecting duct) tumors [2]. Analysis of the SEER database indicates that RCC incidence has been rising on average 0.6% each year and death rates have been falling on average 1.6% each year from 2010 through 2019 [3]. The most important prognostic determinants of 5-year survival are the tumor stage, grade, local extent of the tumor, presence of regional nodal metastases, and evidence of metastatic disease at presentation. RCC primarily metastasizes to the lung, bone, liver, lymph nodes, adrenal gland, and brain. Clinical characteristics of patients have been extensively analyzed as potential prognostic factors in metastatic renal cell cancer (RCC). A combination of these variables has been used to stratify patients into “risk groups” to predict outcomes, to segregate patients for randomized clinical trial entry, and to aid in the interpretation of nonrandomized clinical trials. The most common schemas from the Memorial Sloan Kettering Cancer Center was developed from patients treated with interferon-based regimens and International Metastatic Database Consortium (IMDC) which consists of using hemoglobin, corrected calcium, performance status, and time from diagnosis to treatment, but neutrophil and platelet count as well [4,5]. Both Memorial Sloan Kettering Cancer Center and IMDC criteria are used to describe patient populations treated in the targeted therapy era. The most preferred model derived from a population of patients with metastatic RCC treated with vascular endothelial growth factor receptor (VEGFR)-targeted therapy followed the IMDC (International Metastatic RCC Database Consortium) model. Targeted therapy utilizing TKIs, and/or anti-VEGF antibodies has been widely used in first- and second-line treatments. A number of targeted agents has been approved by the FDA for the treatment of advanced RCC in the first and/or subsequent lines of therapy.

VEGFR-targeted therapies have created a new environment for clinical trial development and patient care in patients with metastatic RCC. These targeted therapies, which act as tyrosine kinase inhibitors (TKIs), enhance disease control and improve overall survival, reaching a median OS of about 29 months, and are recommended by international guidelines (sunitinib, pazopanib, etc.) as an adequate therapy agent [6,7]. TKI options have been the standard of care for patients with locally advanced or metastatic renal cell cancer [8]. Novel immunotherapy and TKIs combinations (lenvatinib plus pembrolizumab, cabozantinib plus nivolumab, axitinib plus pembrolizumab, and axitinib plus avelumab) have been found to improve the patient’s prognosis, especially in patients with a poor-risk score based on IMDC score system. The relevance of the IMDC prognostic criteria in the era of frontline combination immunotherapy remains to be established. In the absence of alternative immunotherapy-based prognostic criteria, these criteria continue to be used in clinical trials to risk-stratify patients and, to some extent, by providers and clinical guidelines to direct therapy. Moreover, TKI monotherapy remains an appropriate first-line therapy for a substantial proportion of patients who are not suitable for immunotherapy. The current STAR trial also demonstrated that planned breaks in tyrosine kinase inhibitor treatment in patients with renal cell carcinoma might be a reasonable option when there is a patient or healthcare need (e.g., a pandemic or drug shortage); with caution, this should be exercised since these patients would typically have shorter progression-free survival than those receiving first-line tyrosine kinase inhibitors [9]. In addition, a meta-analysis showed that favorable-risk group patients who have been treated with TKI (sunitinib) have similar overall survival rates compared to immunotherapy plus TKI treatment regimens [10]. On the other hand, the IMDC and Memorial Sloan Kettering Cancer Center models stratify patients with 1 or 2 risk factors as intermediate prognoses. The intermediate-risk score has heterogeneity that may influence the response to TKIs and the prognosis of these patients may be similar to either a poor-risk score or a favorable-risk score [11,12]. There is a need for a better stratification factor to segregate these heterogeneous groups as favorable or unfavorable prognoses. The prognostic nutritional index (PNI) is another prognostic indicator for patients with advanced-stage renal cell carcinoma [13]. A recent meta-analysis has demonstrated that PNI is a good diagnostic accuracy as a prognostic indicator for RCC.

In this study, we aimed to demonstrate whether the adjusted PNI-IMDC risk-score system has a role in stratifying the intermediate group as having favorable or unfavorable prognoses in patients who received TKIs in the first-line setting.

## 2. Methods

### 2.1. Study Subjects

This retrospective study used registry data of 185 metastatic RCC patients who received tyrosine-kinase inhibitors in the first-line setting from 2 medical oncology clinics. The institutional ethics committees approved the studies. Treatment agents were pazopanib (administered 800 mg once daily) and sunitinib (administered according to one of the following schedules: 50 mg once daily for 2 consecutive weeks followed by 1 week or 50 mg once daily for 4 consecutive weeks followed by 2 weeks) based on physician choice and/or patient’s reference.

**Figure 1:**
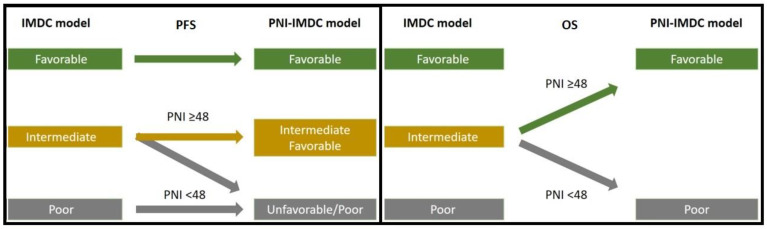
Adjusted PNI-IMDC model for predicting PFS and OS.

The information about the clinicopathological and demographic characteristics of the patients including age, gender, ECOG performance score, histopathology, the IMDC score, and treatment options were collected from medical records. The laboratory findings prior to treatment were included in this study. Complete blood count parameters including neutrophil, lymphocyte, monocyte, and platelet counts were assessed retrospectively. Serum albumin, LDH, and calcium values prior to treatment were also assessed. The same data of hematological and biochemical values for calculating PNI and the IMDC were used. In addition, PNI scores were calculated as serum albumin (g/L) + 5 × total lymphocyte count (10^9^/L). The optimum cut-off value for PNI was 48 based on the receiver operating characteristic (ROC) curve analysis. The patients were divided into two groups based on their initial PNI scores: low PNI (<48) and high PNI (≥48) groups. Then, the IMDC was calculated according to the relevant clinical parameters [14]. These parameters include Karnofsky performance status (<80), time from the initial diagnosis to initiation of targeted therapy (<1 year), hemoglobin level less than the lower limit of normal, serum calcium level greater than the upper limit of normal, neutrophil count greater than the upper limit of normal, and platelet count greater than the upper limit of normal. If none of the above risk factors are grouped as favorable risk, 1 or 2 of the above risk factors are grouped as intermediate risk, and 3 or more risk factors are grouped as poor risk. Finally, the adjusted PNI-IMDC classification model was performed to stratify the intermediate group into 2 groups (unfavorable (PNI < 48) intermediate and favorable (PNI ≥ 48) intermediate groups) for predicting the prognostic effect on both progression-free survival either (PFS) and overall survival (OS) (Figure 1).

### 2.2. Statistical Analysis

Prognostic analysis was calculated based on PFS (defined as the time from the first day of first-line tyrosine kinase inhibitors to the date of disease progression or death) and OS (defined as the time between the diagnosis of metastatic disease and death or date of last known alive). Data analysis was performed using SPSS 22.0 statistical software. We constructed the ROC curve analysis to find the optimal cut-off values for PNI. The area under the ROC curve (AUC) was calculated, and 95% confidence intervals were used to test the hypothesis that the AUC is 0.5. Continuous data were summarized as median and interquartile range. A Chi-square or Fisher’s exact test was used to analyze the categorical variables. Survival curves were obtained using the Kaplan–Meier method for each subgroup, with 95% confidence intervals (CIs) calculated using the Brookmeyer and Crowley method, and the differences in survival between the groups were compared by log-rank test. The univariate analysis was used to examine the prognostic importance of any factors. Prognostic factors with a *p*-value of <0.5 in the univariate analysis were examined in the multivariate analysis. Hazard ratios (HRs) for these comparisons were calculated using a Cox proportional hazards model. *p* < 0.05 was considered statistically significant.

## 3. Results

### 3.1. Study Patients

**Figure 2:**
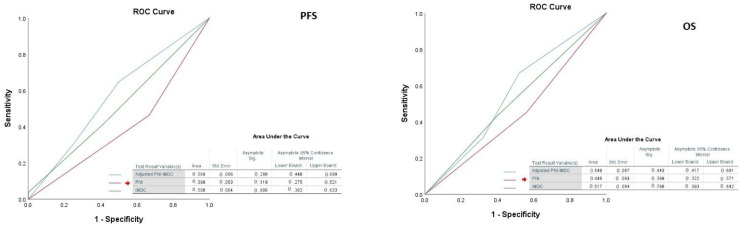
ROC curve analysis of the models in regard to both PFS and OS.

The median age was 61 (51–69) and male to female ratio was 2.62. Of these, 18.2% had non-clear cell histology. IMDC score consisted of favorable (47%), intermediate (33.4%), and poor (3.9%). Seventy-nine percent of the patients had prior nephrectomy and only 53.2% had lower PNI (<48). About 85.6% had visceral metastasis which consisted of (79.0%) of the lung, (17.7%) of the liver, and (9.4) brain metastasis. For the first-line setting, about 67.4% of them received sunitinib, and the remaining received pazopanib. The cut-off PNI value to divide the two groups (PNI < 48 and PNI ≥ 48) was determined after ROC curve analysis. C-index analysis showed that PNI, classical IMDC, and adjusted PNI-IMDC risk score models had comparable prognostic prediction in regard to both PFS and OS (Figure 2).

### 3.2. Univariate and Multivariate Analysis for Progression-Free Survival

Table 1 shows the univariate analysis for PFS of first-line tyrosine kinase inhibitors. Median PFS was 7.0 (95% CI 4.5–9.3) months. The first-line median PFS value of sunitinib was comparable to that of pazopanib (*p* = 0.78). The presence of liver metastasis was associated with lower PFS value (HR 1.725 (1.069–2.784, *p* = 0.02) and patients who had metastasectomy had significantly longer PFS value (HR 0.616 (0.398–0.952), *p* = 0.03). According to the IMDC risk score, the favorable group had the longest PFS (20.4 (16.6–24.2)) value rather than PFS values of both intermediate (6.4 (4.8–8.0)) and poor (3.8 (2.9–4.7)) risk groups (*p* < 0.001). In addition, patients who had lower PNI had lower PFS values than patients who had higher PNI values (3.8 (3.3–4.2) vs. 10.9 (7.7–14.0), *p* < 0.001, respectively). On the other hand, according to the adjusted PNI and IMDC risk score intermediate (PNI-low) group had a similar PFS value compared to that of the poor-risk group (HR 1.027 95%CI 0.521–2.025, *p* = 0.93).

**Figure 3:**
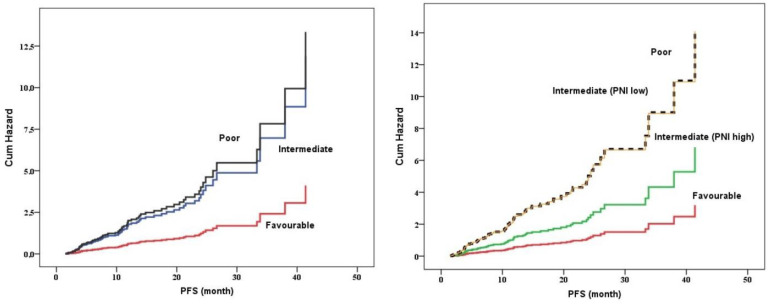
Kaplan–Meier curve for PFS based on classic IMDC and adjusted PNI-IMDC model.

Table 2 shows the multivariate analysis for PFS of first-line tyrosine kinase inhibitors. Model 1 including histology, adjusted PNI-IMDC risk score model, the presence of metastasectomy, and the presence of liver metastasis demonstrated that adjusted PNI and IMDC risk scores are significantly independent risk factors for PFS of first-line tyrosine kinase inhibitors. On the other hand, model 2 including PNI and IMDC risk score, presence of metastasectomy, and presence of liver metastasis demonstrated that both PNI and IMDC risk score significantly independent risk factors for PFS of first-line tyrosine kinase inhibitors. Adjusted PNI and IMDC model demonstrated that there was a separate group which had a PNI-low value (<48) in the intermediate group according to classic IMDC was associated with a shorter PFS value as similar to the poor-risk group according to IMDC (Figure 3).

### 3.3. Univariate and Multivariate Analysis for Overall Survival

Table 1 shows the univariate analysis for overall survival. Median OS was 21.2 (95% CI 13.0–29.4) months. Univariate analysis demonstrated that high ECOG performance score (*p* < 0.001), poor IMDC score (*p* = 0.004), low PNI (*p* = 0.001), and adjusted PNI and IMDC model (poor (*p* = 0.001) and intermediate PNI-low (*p* = 0.01)), absence of primary nephrectomy (*p* < 0.001), presence of bone metastasis (*p* = 0.007), presence of liver metastasis (*p* = 0.03), and 3 and more metastatic sites (*p* = 0.007) were significantly associated with poor prognosis, respectively.

**Figure 4:**
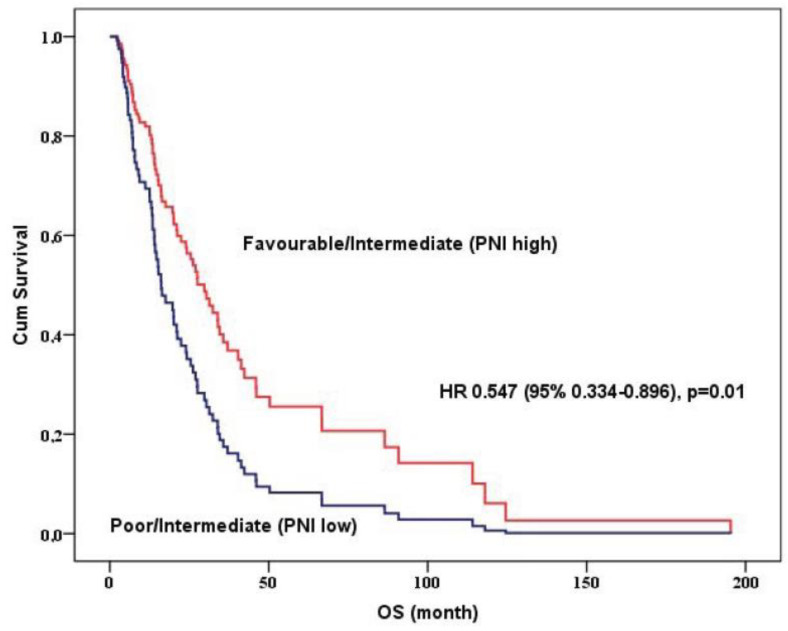
Overall survival curve using Cox regression analysis according to adjusted PNI-IMDC model.

Table 3 shows the multivariate analysis for overall survival. Multivariate analysis demonstrated that the presence of nephrectomy (*p* = 0.002), adjusted favorable or intermediate (PNI-high) group (*p* = 0.01), ECOG performance score (*p* = 0.001), and presence of bone metastasis (*p* = 0.004) were independent predictors of OS, respectively. Adjusted PNI and IMDC model was divided into 2 groups favorable/intermediate PNI-high and poor/intermediate PNI-low groups and the favorable/intermediate PNI-high group was significantly associated with a more favorable prognosis rather than poor/intermediate PNI-low groups (*p* = 0.01, Figure 4)

## 4. Discussion

In this study, we determined that the pre-treatment adjusted PNI-IMDC classification model was an independent prognostic indicator in mRCC patients who received TKIs in the first-line setting. Our adjusted PNI-IMDC classification model markedly and clearly identified these patients into two groups to categorize as unfavorable (PNI < 48) intermediate and favorable (PNI ≥ 48) intermediate groups. It was demonstrated that patients with unfavorable (PNI < 48) intermediate-risk scores had comparable prognoses to those with poor-risk scores based on the classical IMDC score model. On the other hand, patients with favorable (PNI ≥ 48) intermediate-risk scores had comparable prognoses to those with favorable-risk scores based on the classical IMDC score model. Hence, the pre-treatment adjusted PNI-IMDC classification model is useful for establishing a more improved prognostic model that is able to stratify mRCC patients treated with first-line TKIs. To the best of our knowledge, this is the first study assessing PNI as a potential add-on biomarker to improve the IMDC prognostic score in patients with metastatic RCC intermediate group by segregating into favorable and poor-risk groups based on their adjusted PNI-IMDC model score.

The choice of treatment for patients with advanced clear cell RCC has been based on prognostic risk factors historically developed in the era of frontline VEGFR-TKIs. The treatment approach to patients with metastatic non-clear cell RCC is varied and, to some extent, tailored to the histologic subtype and pathologic and molecular features of the tumor. The main histologic subtypes of non-clear cell RCC include papillary, chromophobe, collecting duct (including medullary carcinoma), translocation, and unclassified. Although many advances have been made in the treatment of metastatic non-clear cell RCC, there are limited high-quality data to help inform management, due to the infrequency of these tumors. Mostly, VEGFR-TKIs have been used as front-line settings like those in clear cell RCC. In addition, sarcomatoid features, or sarcomatoid RCC, are not considered a distinct subtype of RCC because sarcomatoid features can be seen in any histologic subtype of RCC, including both clear cell and non-clear cell histologies. Advanced or metastatic sarcomatoid RCC is clinically responsive to immunotherapy-based regimens. Combinations of immunotherapy plus antiangiogenic therapy are active in patients with advanced or metastatic RCC. Current treatment guidelines recommend that most treatment-naive patients with advanced or metastatic clear cell RCC receive systemic therapy with molecularly targeted therapy and/or immunotherapy based on risk factors and/or disease burden [15].

The introduction of molecular targeted agents into advanced-stage renal cell carcinoma therapy led to improved survival rates. In order to stratify the mRCC patients for predicting prognosis and enhancing more appropriate and effective treatment decision-making in the first-line setting, some stratification tools such as MSKCC and IMDC have been used in daily oncology routine practice for several years. First, MKSCC had been developed in the pre-targeted therapy era. Then, IMDC classification was developed in the molecular-targeted therapy era [4,14,16]. These models were based on several hematological and clinical features allocating patients into three groups to direct the physician guiding more appropriate choice of treatment for patients with advanced disease in the era of frontline TKIs. Due to fact the absence of alternative immunotherapy-based prognostic criteria, IMDC prognostic criteria remain to be established in clinical trials to stratify the patients and clinical guidelines to guide the therapy (NCCN guidelines, ESMO guidelines) as well. On the other hand, it was demonstrated that PNI has value as a prognostic factor for patients with advanced RCC [13]. Hence, there is a strong inverse relationship between the PNI and tumor aggressiveness, and a lower PNI was also associated with poorer patient outcomes [17]. This suggests that there is a potential marker for a significant association among the PNI, pathological characteristics of RCC, and other known risk factors for patients with advanced RCC [13]. Notably, the IMDC intermediate-risk group is highly heterogeneous, and there have been attempts to improve the prognostic stratification of these patients by considering the number of IMDC prognostic factors [18]. Despite recent advances in the treatment of patients with advanced RCC, the prognosis of the IDMC intermediate-risk group remains challenging. Although current guidelines recommend first-line treatment as same as a poor-risk group, some patients in the intermediate group may have similar prognosis to patients in the favourable group. Several studies have attempted to identify additional factors that can precisely predict the prognosis of RCC [19]. Therefore, the choice of therapy regimens for intermediate-risk groups remains controversial. Using the PNI together with IMDC score variables to stratify intermediate-risk patients into two-risk groups has been shown to segregate intermediate-risk patients into two groups who were as similar outcomes as the poor-risk group and favourable-risk group. In this respect, we suggest that the pre-treatment adjusted PNI-IMDC classification model significantly contributes to better stratification of the intermediate according to their prognosis and better predicting the TKIS response in the pretreatment period.

Multiple prognostic factors have been established or underestimated, including tumor size, presence of sarcomatoid differentiation, performance status, liver, bone, and lymph node metastasis, and a number of metastatic sites [20,21]. Low-performance status is a measure of overall well-being and is the most consistently reported factor associated with survival in advanced RCC [22]. In addition, some studies have revealed that the presence of bone metastasis, brain metastasis, and visceral metastasis (liver, lung, etc.) is related to poor prognosis, whereas some studies revealed no relationship between these sites and prognosis [23]. A more reliable variable is the number of the presence of metastatic sites, which correlates with the tumor burden. Several studies have found that patients with a higher number of metastatic sites (>2) are independently associated with at least a two-fold greater probability of death [24,25]. On the other hand, the presence of asynchronous metastasis and primary nephrectomy have been found to be associated with prognosis [26]. The role of primary nephrectomy in metastatic RCC is still debatable, it was speculated that removing the large primary tumor could have had an impact on prognosis. This hypothesis is in concordance with the European Society for Medical Oncology (ESMO) guidelines recommending CN in the presence of large primary tumors [27]. Similarly, we found that low-performance status, presence of bone and liver metastasis, a higher number of metastases, and absence of primary nephrectomy were associated with poor prognosis.

There are several active agents now exist for metastatic RCC, unfortunately, they are noncurative for most patients and thus need chronic treatment. The treatment benefit must be weighed against the toxicity, time commitment, and cost [28]. In addition, the inhibition of multiple targets by the TKIs can lead to various adverse events, among which hematologic and hepatic toxicities are particularly significant and necessitate careful monitoring [29]. However, there is still no definitive evidence to support a relationship between the severity of adverse events and efficacy. Although these toxicity profiles, most of the adverse events are tolerable and tyrosine kinase inhibitors alone are one of the preferred options of patients in the favorable-risk group, and the immunotherapy plus tyrosine kinase inhibitor combination is also another option for both favorable and intermediate/poor-risk groups. VEGFR and TKIs also have demonstrated substantial anti-tumor activity as a second-line therapy in patients with metastatic RCC who progressed on cytokine therapy. In our country, regardless of risk stratification, we use tyrosine kinase in the first-line setting. Here, we adjusted the PNI-IMDC classification model on the Turkish patients with mRCC treated with first-line tyrosine kinase inhibitors, confirming its prognostic value in this treatment setting, alongside a higher accuracy than the IMDC alone.

There are some major limitations. First, retrospective clinical data of the patients from medical records has disadvantages in controlling for all potential confounding biases that may influence the prognosis. Second, the number of patients was small, which hampered the present results to be applied to all mRCC patients. Then, data about toxicity profile was not considered due to missing data leading to incomplete identification of adverse events considering the limitation of this retrospective study. Despite these limitations, this is the first study assessing PNI as a potential add-on biomarker to improve the IMDC prognostic score in patients with metastatic RCC intermediate group by segregating them into favorable and poor-risk groups based on their adjusted PNI-IMDC model score.

In conclusion, the present study demonstrated that pre-treatment PNI as a valuable and potential add-on biomarker to the adjusted PNI-IMDC classification model is useful for establishing an improved prognostic model in mRCC patients treated with first-line TKIS. Further validation studies are needed to clarify these findings.

## Figures and Tables

**Table 1:** Univariate analysis for predicting progression-free survival and overall survival.

	Progression-Free Survival			Overall Survival		
	Median (95%CI)	HR (95% CI)	*p*-Value	Median (95%CI)	HR (95% CI)	*p*-Value
Age						
<60	6.4 (3.6–9.2)	Ref.	0.92	21.9 (2.1–40.2)	Ref.	0.3
≥60	7.7 (4.6–10.8)	1.023 (0.633–1.652)		22.3 (12.2–32.5)	1.264 (0.811–1.970)	
Gender						
Female	6.4 (2.8–9.9)	Ref.	0.41	17.5 (9.1–25.9)	Ref.	0.12
Male	6.9 (5.1–8.6)	0.826 (0.523–1.305)		25.4 (17.7–33.1)	0.684 (0.423–1.107)	
ECOG performance score						
0–1	7.2 (5.6–8.7)	Ref.	0.11	25.4 (19.1–31.7)	Ref.	<0.001
≥2	3.5 (2.8–4.2)	2.096 (0.844–5.206)		4.1 (3.1–5.1)	7.657 (3.529–16.612)	
Histopathology						
Clear cell	7.4 (5.8–9.0)	Ref.	0.4	23.9 (14.4–33.4)	Ref.	0.85
Non-clear cell	4.3 (2.4–6.1)	1.233 (0.753–2.018)		19.7 (9.0–30.4)	1.051 (0.606–1.824)	
IMDC score, n (%)						
Favorable	20.4 (16.6–24.2)	Ref.	<0.001	41.3 (0.5–82.1)	Ref.	0.004
Intermediate	6.4 (4.8–8.0)	3.698 (1.893–7.226)	<0.001	20.1 (10.4–29.7)	1.717 (0.959–3.074)	0.07
Poor	3.8 (2.9–4.7)	6.201 (2.824–13.615)	<0.001	12.4 (1.2–23.7)	3.411 (1.641–7.089)	0.001
PNI						
<48	3.8 (3.3–4.2)	Ref.	<0.001	12.5 (5.5–19.5)	Ref.	0.001
≥48	10.9 (7.7–14.0)	0.444 (0.298–0.662)		32.3 (17.2–47.5)	0.460 (0.291–0.726)	
Adjusted IMDCC and PNI model						
Favorable	20.4 (16.6–24.2)	Ref.	<0.001	41.3 (0.5–82.1)	Ref.	0.001
Intermediate (PNI-High)	10.8 (8.8–12.8)	2.648 (1.302–5.386)	0.007	30.3 (18.0–42.6)	1.219 (0622–2.390)	0.564
Intermediate (PNI-Low)	3.8 (3.6–3.9)	5.497 (2.722–11.103)	<0.001	12.5 (5.6–19.4)	2.315 (1.223–4.382)	0.01
Poor	3.8 (2.9–4.7)	6.108 (2.803–13.311)	<0.001	12.4 (1.2–23.7)	3.510 (1.685–7.312)	0.001
Primary nephrectomy, n (%)						
Absent	3.7 (2.6–4.7)	Ref.	0.85	7.8 (4.5–11.1)	Ref.	<0.001
Present	7.7 (6.2–9.1)	0.955 (0.571–1.595)		30.3 (21.0–39.6)	0.366 (0.228–0.588)	
Sarcomatoid differentiation, n (%)						
Absent	7.1 (4.0–10.3)	Ref.	0.59	37.0 (26.8–47.2)	Ref.	0.08
Present	7.5 (6.5–8.5)	0.849 (0.465–1.550)		15.1 (11.1–19.1)	2.097 (0.913–4.815)	
Metastasectomy, present	11.6 (4.5–18.8)	0.616 (0.398–0.952)	0.03	27.5 (15.4–39.6)	0.728 (0.446–1.187)	0.2
Lung, present	11.7 (3.0–20.5)	0.707 (0.325–1.540)	0.38	34.5 (25.3–43.7)	0.570 (0.224–1.451)	0.23
Bone, present	6.4 (2.8–10.0)	1.912 (0.828–4.418)	0.12	20.0 (2.7–37.3)	1.496 (0.552–4.056)	0.42
Metastatic site						
Bone, present	4.3 (2.1–6.4)	1.01 (0.677–1.481)	0.99	15.1 (12.0–18.3)	1.865 (1.190–2.923)	0.007
Visceral, present	6.4 (4.4–8.4)	1.192 (0.599–2.368)	0.61	20.1 (12.2–27.9)	1.597 (0.768–3.320)	0.21
Visceral metastasis						
Lung, present	6.8 (5.1–8.5)	0.934 (0.548–1.593)	0.8	22.3 (12.1–32.6)	1.183 (0.663–2.109)	0.56
Liver, present	4.3 (1.8–6.7)	1.725 (1.069–2.784)	0.02	13.2 (10.0–16.4)	1.710 (1.032–2.831)	0.03
Brain, present	12.4 (6.9–17.9)	0.632 (0.318–1.258)	0.19	16.0 (6.6–25.4)	1.184 (0.569–2.466)	0.65
Metastasis site number						
<3	7.4 (5.4–9.4)	Ref.	0.36	32.3 (16.0–48.6)	Ref.	0.007
≥3	4.2 (1.3–7.2)	1.208 (0.801–1.819)		15.1 (7.1–23.2)	1.831 (1.177–2.851)	
First-line tyrosine kinase option						
Sunitinib	6.0 (3.9–8.0)	Ref.	0.78	25.4 (16.7–34.1)	Ref.	0.21
Pazopanib	7.3 (3.6–11.1)	0.944 (0.618–1.441)		15.2 (12.5–18.0)	1.325 (0.846–2.076)	

**Table 2:** Multivariate analysis for predicting progression-free survival.

	Model 1		Model 2	
	HR (95% CI)	*p*-Value	HR (95% CI)	*p*-Value
Histopathology, clear cell	0.660 (0.380–1.145)	0.13	0.652 (0.378–1.126)	0.12
IMDC score				0.008
Favorable			0.308 (0.133–0.714)	0.006
Intermediate			0.890 (0.508–1.561)	0.68
Poor			Ref.	
PNI ≥ 48			0.503 (0.328–0.773)	0.002
Adjusted IMDCC and PNI model		<0.001		
Favorable	0.226 (0.099–0.516)	<0.001		
Intermediate (PNI-High)	0.484 (0.270–0.866)	0.01		
Intermediate (PNI-Low)	1.006 (0.556–1.822)	0.98		
Poor	Ref.			
Metastasectomy, present	0.680 (0.433–1.068)	0.09	0.683 (0.437–1.067)	0.09
Liver, present	1.436 (0.859–2.401)	0.16	1.518 (0.909–2.533)	0.11

**Table 3:** Multivariate Cox regression analysis for overall survival.

	HR (95% CI)	*p*-Value
Nephrectomy, present	0.449 (0.270–0.748)	0.002
Adjusted IMDCC and PNI model		0.01
Favorable/Intermediate (PNI-High)	0.547 (0.334–0.896)	
ECOG 2 and more, *present*	4.220 (1.815–9.814)	0.001
Bone metastasis, *present*	1.997 (1.252–3.185)	0.004

## Data Availability

Data available on request.
